# ACE Reduces Metabolic Abnormalities in a High-Fat Diet Mouse Model

**DOI:** 10.1155/2015/352647

**Published:** 2015-10-05

**Authors:** Seong-Jong Lee, Jong-Min Han, Jin-Seok Lee, Chang-Gue Son, Hwi-Jin Im, Hyun-Kyung Jo, Ho-Ryong Yoo, Yoon-Sik Kim, In-Chan Seol

**Affiliations:** ^1^Internal Medicine of Cardiac Vascular System, Daejeon Oriental Hospital of Daejeon University, 176-75 Daedeok-daero, Seo-gu, Daejeon 302-869, Republic of Korea; ^2^Liver and Immunology Research Center, Daejeon Oriental Hospital of Daejeon University, 176-9 Daeheung-ro, Jung-gu, Daejeon 301-724, Republic of Korea

## Abstract

The medicinal plants *Artemisia iwayomogi* (*A. iwayomogi*) and *Curcuma longa* (*C. longa*) radix have been used to treat metabolic abnormalities in traditional Korean medicine and traditional Chinese medicine (TKM and TCM). In this study we evaluated the effect of the water extract of a mixture of *A. iwayomogi* and *C. longa* (ACE) on high-fat diet-induced metabolic syndrome in a mouse model. Four groups of C57BL/6N male mice (except for the naive group) were fed a high-fat diet freely for 10 weeks. Among these, three groups (except the control group) were administered a high-fat diet supplemented with ACE (100 or 200 mg/kg) or curcumin (50 mg/kg). Body weight, accumulation of adipose tissues in abdomen and size of adipocytes, serum lipid profiles, hepatic steatosis, and oxidative stress markers were analyzed. ACE significantly reduced the body and peritoneal adipose tissue weights, serum lipid profiles (total cholesterol and triglycerides), glucose levels, hepatic lipid accumulation, and oxidative stress markers. ACE normalized lipid synthesis-associated gene expressions (peroxisome proliferator-activated receptor gamma, PPAR*γ*; fatty acid synthase, FAS; sterol regulatory element-binding transcription factor-1c, SREBP-1c; and peroxisome proliferator-activated receptor alpha, PPAR*α*). The results from this study suggest that ACE has the pharmaceutical potential reducing the metabolic abnormalities in an animal model.

## 1. Introduction

Metabolic syndrome has become a serious health issue worldwide as the prevalence of obesity increases [1]. A previous cohort study reported a strong relationship between metabolic syndrome and an increased risk of mortality [2]. Metabolic syndrome is defined as a cluster of metabolic abnormalities including obesity, hyperlipidemia, hyperglycemia, insulin resistance, and hypertension [3]. Generally, an individual is considered to have metabolic syndrome if he/she has central obesity plus any two of the above factors [1]. Improving these pathological conditions, especially obesity, is major therapeutic goals for treating metabolic syndrome.

Lifestyle modifications, including exercise and diet control, are recommended as a first-line treatment for managing metabolic syndrome [3]. However, drug therapy is now also considered an attractive method of treating metabolic syndrome. Various agents are currently used specifically for lowering lipid profiles or blood pressure, but novel therapies targeting multiple etiological factors are particularly desirable [4]. Natural remedies are currently attracting more attention as therapeutic or protective agents for treating metabolic syndrome [5].

In traditional Korean medicine (TKM) and traditional Chinese medicine (TCM), metabolic abnormalities are considered indicative of “*dampness/phlegm*” (*濕痰*) and “*blood stasis*” (*瘀血*) [6].* Artemisia iwayomogi* (*A. iwayomogi*) is a representative herb treating dampness/phlegm in TKM and TCM. Previous studies have reported the therapeutic properties of* A. iwayomogi *on obesity, hyperlipidemia, and liver fibrosis [7, 8]. Conversely,* Curcuma longa *(*C. longa*) has been used to treat the pathological condition of blood stasis and reportedly exerts a beneficial effect on hyperlipidemia [9, 10]. Based on the long-term clinical experience and prestudies for the synergic effects using various combinations of herbs, the formula, the mixture of* A. iwayomogi and C. longa*, was finalized. However, to date, no study has evidenced the combined effect of* A. iwayomogi *and* C. longa* (ACE) on metabolic syndrome.

Therefore, we evaluated the effect of ACE on major manifestations of metabolic syndrome including obesity, hyperlipidemia, and fatty liver using a high-fat diet mouse model.

## 2. Materials and Methods

### 2.1. Preparation and Fingerprinting of ACE

*A. iwayomogi *and* C. longa *were purchased from a traditional medicine store (Jeong-Seong Drugstore, Daejeon, Korea). To produce ACE, 250 g of each herb was mixed and boiled in 2 L of distilled water for 90 min using an automatic nonpressure pot (Dae-Woong, Seoul, Korea). After filtering using a 300-mesh filter and inspiration for 60 min, the extract was centrifuged for 15 min at 150 ×g. Finally, the supernatant was lyophilized using a vacuum freeze-drying system and stored at −20°C. The extraction yield was 12.59%.

**Figure 1:**
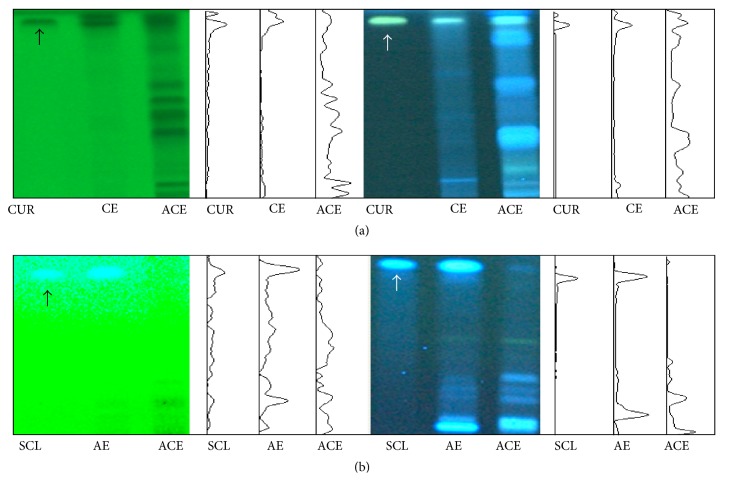
Fingerprint analysis of ACE using high-performance thin-layer chromatography (HP-TLC). ACE and its two major components were analyzed using HP-TLC and compared with reference compounds. Scopoletin (SCL, 0.1 *μ*g/*μ*L (a)),* Artemisia iwayomogi* (AI, 10 *μ*g/*μ*L (a)), curcumin (CUR, 0.1 *μ*g/*μ*L (b)),* Curcuma longa* radix (CL, 10 *μ*g/*μ*L (b)), and ACE (10 *μ*g/*μ*L ((a) and (b))) were applied to prewashed silica gel 60 F254 TLC plates and then separated in the mobile phase (chloroform : ethyl acetate : methanol : water = 17 : 46 : 25 : 12). The migrated components were visualized under UV light at 254 nm (left) or 366 nm (right).

To confirm the reproducibility of the extraction procedure, a high-performance thin-layer chromatography- (HP-TLC-) based fingerprint was produced using the CAMAG sample application technique (Muttenz, Switzerland, Figure 1) [11].

### 2.2. Chemicals and Reagents

The following reagents were purchased from Sigma Aldrich (St. Louis, MO, USA): 7-hydroxy-6-methoxycoumarin (scopoletin), curcumin, p-dimethylaminobenzaldehyde, 1,1,3,3-tetraethoxypropane (TEP), chloramines-T, 5,5-dithiobis-2-nitrobenzoic acid (DTNB), reduced glutathione, glutathione reductase (GSH-Rd), glutathione peroxidase (GSH-Px), *β*-nicotinamide adenine dinucleotide phosphate (*β*-NADP), and *β*-NADPH. Perchloric acid was obtained from GFS Chemical Co. (Columbus, OH, USA), thiobarbituric acid (TBA) from Lancaster Co. (Lancashire, England, UK), and hydrogen peroxide from Junsei Chemical Co., Ltd. (Tokyo, Japan).

### 2.3. Animals and Experimental Design

Fifty specific pathogen-free C57BL/6N male mice (6 weeks old, 22–24 g) were obtained from Koatech (Gyeonggi-do, Korea). The mice had free access to pelleted food (Koatech, Gyeonggi-do, Korea) and water and were housed in a room with a temperature maintained at 23 ± 2°C and under a 12:12-h light-dark cycle. After a one-week acclimatization period, mice were randomly divided into five groups: naive group (*n* = 10, AIN-76, Dyets Inc., Bethlehem, PA, USA), control group (*n* = 10, 60% high-fat diet, D124912, Research Diets, Inc., New Brunswick, NJ, USA), ACE groups (*n* = 10, 60% high-fat diet with 100 or 200 mg/kg ACE), and curcumin group (*n* = 10, 60% high-fat diet with curcumin 50 mg/kg). ACE and curcumin were mixed into the high-fat diet and the quantity of drug was determined by calculating the daily feeding amount (approximately 3 g/day); 1.10 g or 2.20 g ACE; and 0.55 g curcumin per 1 kg high-fat diet.

This animal experiment was approved by the Institutional Animal Care and Use Committee of Daejeon University (DJUARB2012003) and was conducted in accordance with the Guide for the Care and Use of Laboratory Animals published by the U.S. National Institutes of Health (Bethesda, MD, USA).

### 2.4. Measurement of Food Intake and Body, Liver, and Adipose Tissue Weights

Food intake and body weight were monitored weekly. Mice were euthanized using ether on the final day of experiment after a 12-h fast and whole blood was collected* via* the abdominal aorta. The liver and adipose tissues (epididymal, retroperitoneal, and visceral) were removed, weighed, and frozen in liquid N2 or stored in RNAlater (Qiagen, Valencia, CA, USA).

### 2.5. Histopathological Analysis

For the histopathological evaluation, freshly isolated liver and white adipose tissues (epididymal and retroperitoneal) were fixed in 10% formalin for 24 h. Following sufficient rinsing in flowing water, tissues were processed in a paraffin automatic processor using a programmed cascade. The paraffin-embedded samples were dissected into 4-*μ*m thick sections and stained with hematoxylin and eosin (H&E). After H&E staining of the liver and adipose tissues, representative histopathological features such as steatosis and adiposity were observed under a microscope. After one photograph per sample was obtained for the stained adipose tissue using an optical microscope operating at magnifications of ×200 and ×400, the size of 10 randomly selected adipocytes per photograph was measured using a computer image analysis program (NIH, USA) to obtain average values. For immunohistochemistry against 4-hydroxynonenal (4-HNE), sections were incubated with 4-HNE primary antibody (1 : 200; Abcam, Cambridge, UK) and biotinylated secondary antibody (Nichirei Biosciences, Tokyo, Japan), followed by avidin-biotin-peroxidase complex. The immunoreactive signal was developed using its substrates, AEC (Abcam). The slides were counterstained with Mayer's hematoxylin (Sigma Aldrich) and examined under an optical microscope (Leica Microsystems, Wetzlar, Germany).

### 2.6. Determination of Lipid Levels in Liver Tissue

Livers were homogenized in PBS and protein concentrations determined. Then, 300 *μ*L of homogenate was extracted with 5 mL of chloroform/methanol (2 : 1) and 0.5 mL of 0.1% sulfuric acid [12]. An aliquot of the organic phase was collected, dried under nitrogen, and resuspended in 2% Triton X-100. Hepatic triglyceride content was determined using commercially available kits. Data were normalized for differences in protein concentration.

### 2.7. Serum Lipid Profiles and Glucose

Serum levels of total cholesterol (TC), low-density lipoprotein cholesterol (LDL-C), high-density lipoprotein cholesterol (HDL-C), triglycerides, and glucose were determined using an autoanalyzer (Chiron, Emeryville, CA, USA).

### 2.8. Determination of Reactive Oxygen Species (ROS) in Serum and Liver Tissues

The total amount of ROS in the serum or liver tissue samples was determined using a method described previously [13]. The amount of ROS was determined at 505 nm using a spectrophotometer (Molecular Devices Corp., Sunnyvale, CA, USA).

Radioimmune precipitation assay (RIPA) buffer-based liver tissue homogenates were centrifuged at 10,000 ×g for 15 min. The supernatants were transferred to clean tubes and stored at −70°C until required. The protein concentration was determined using the bicinchoninic acid (BCA) protein assay (Sigma Aldrich).

### 2.9. Determination of Lipid Peroxidation (Malondialdehyde, MDA) in Liver Tissue

Lipid peroxidation levels in the liver tissues were evaluated using the thiobarbituric acid reactive substances (TBARS) assay as described previously [14]. The absorbance was measured at 535 and 520 nm using a spectrophotometer (Cary 50; Varian, Palo Alto, CA, USA) and compared with the value from a freshly prepared 1.1.3.3-tetraethoxypropane (TEP) standard.

### 2.10. Determination of Protein Carbonyl Contents in Liver Tissue

Protein carbonyl contents in liver tissue were determined according to the manufacturer's protocol [15]. The absorbance at 370 nm was measured using a spectrophotometer (Molecular Devices Corp.).

### 2.11. Determination of Total Antioxidant Capacity (TAC) in Liver Tissue

TAC levels in the liver tissue were determined as previously described [16]. The absorbance was measured at 600 nm using a spectrophotometer (Molecular Device Corp). TAC was expressed as gallic acid equivalent antioxidant capacity (GEAC).

### 2.12. Determination of Total Glutathione (GSH) Content, GSH-Reductase (GSH-Rd), and GSH-Peroxidase (GSH-Px) in Liver Tissue

Total GSH content and GSH-Rd activity were determined as previously described [17, 18]. The absorbance was measured at 405 nm or 412 nm using a spectrophotometer (Molecular Device Corp.). GSH-Px activity was determined according to a previous method [19]. The final absorbance was measured at 340 nm using a UV-visible spectrophotometer (Varian, Agilent Technologies, Santa Clara, CA, USA).

### 2.13. Determination of Superoxide Dismutase (SOD) and Catalase in Liver Tissue

SOD activity was determined using an SOD assay kit according to the manufacturer's protocol (Dojindo Laboratories, Kumamoto, Japan). The standard concentration was serially diluted from 100 to 0.01 U/mL of bovine erythrocyte SOD (Sigma Aldrich).

Catalase activity was determined as previously described [20]. The absorbance of the purple formaldehyde adduct was measured at 550 nm using a spectrophotometer (Molecular Devices Corp.).

### 2.14. Gene Expression Analysis Using Real-Time Polymerase Chain Reaction (qPCR)

Total RNA was extracted from liver tissue samples with Trizol reagent (Molecular Research Center, Cincinnati, OH, USA). cDNA was synthesized from total RNA (2 *μ*g) in a 20-*μ*L reaction using a High-Capacity cDNA Reverse Transcription Kit (Ambion, Austin, TX, USA). Real-time polymerase chain reaction (qPCR) was performed using SYBRGreen PCR Master Mix (Applied Biosystems, Foster City, CA, USA) and qPCR amplification was performed using a standard protocol with the IQ5 PCR Thermal Cycler (Bio-Rad, Hercules, CA, USA). The following primers (forward and reverse) were used: PPAR-*γ* (NM_011146), 5′-TGG GAG ATT CTC CTG TTG AC-3′, and 5′-AGG TGG AGA TGC AGG TTC TA-3′; SREBP-1c (NM_011480), 5′-GAG CGA GCG TTG AAC TGT A-3′, and 5′-ACT TCA ACG ATG GGG ACT TG-3′; FAS (NM_007988), 5′-TGT GAG TGG TTC AGA GGC AT-3′, and 5′-TTC TGT AGT GCC AGC AAG CT-3′; PPAR-*α* (NM_011144), 5′-CCT GAA CAT CGA GTG TCG AA-3′, and 5′-GTA CTG GCA TTT GTT CCG GT-3′; and *β*-actin (NM_007393), 5′-AGG CTG TGC TGT CCC TGT ATG-3′, and 5′-TGG CGT GAG GGA GAG CAT-3′.

### 2.15. Statistical Analyses

The results are expressed as mean ± standard deviation (SD). The statistical significance of differences between groups was analyzed using one-way analysis of variance (ANOVA), followed by Fisher's least-significant difference (LSD) test. In all analyses, *P* < 0.05 was considered to indicate statistical significance.

## 3. Results

### 3.1. Food Intake and Body, Liver, and Adipose Tissue Weights

Food intake did not differ significantly between the control and naive groups but food intake in the ACE 100 group was significantly increased compared with the control group (*P* < 0.01, Table 1).

After a 10-week high-fat diet, the average body weight in the control group was 1.5-fold heavier compared with the naive group. Administration of ACE significantly reduced the increase of body weight compared with the control group (*P* < 0.001 for both 100 and 200 mg/kg, Table 1).

The absolute and relative liver weights were significantly higher in the control group compared with the naive group. The increase in liver weight was significantly less in the ACE-treated groups than the control group (*P* < 0.05 or *P* < 0.01, Table 1).

The weights of epididymal, retroperitoneal, and visceral adipose tissues were significantly increased (3.5-, 2.5-, and 2.7-fold, resp.) in the control group compared with the naive group. These increases in regional adipose tissues were reduced significantly with ACE 100 mg/kg (*P* < 0.05 in epididymal and total tissues) and ACE 200 mg/kg (*P* < 0.05 in epididymal and retroperitoneal tissues; *P* < 0.01 in total tissue, Table 1). Curcumin had a similar effect on body, liver, and adipose tissue weights.

### 3.2. Histopathological Analysis of Adipose Tissue and Liver

**Figure 2:**
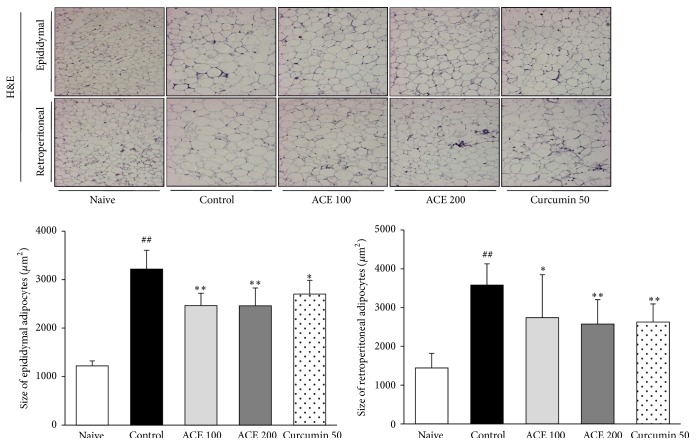
Histological findings of adipose tissues. Epididymal and retroperitoneal tissues were evaluated using hematoxylin and eosin (H&E) staining. All photographs are at ×200 magnification. Cell sizes of adipose tissues were quantified using computer image analysis. ^##^ *P* < 0.01 compared with naive group; ^*∗*^ *P* < 0.05, ^*∗∗*^ *P* < 0.01 compared with control group.

Histological examination of epididymal and retroperitoneal adipose tissues revealed that the adipocyte size markedly increased by 2.5-fold in the control group and that ACE administration significantly reduced these increases (*P* < 0.05 or *P* < 0.01, Figure 2).

**Figure 3:**
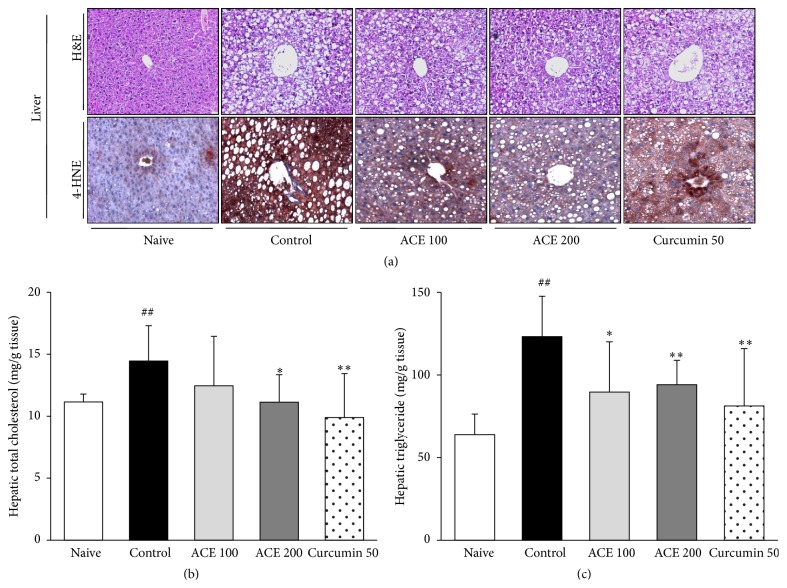
Histopathological findings and lipid profiles of liver tissue. (a) Hepatic tissues were evaluated using hematoxylin and eosin (H&E) staining (upper) and immunohistochemistry for 4-HNE (bottom). All photos are at ×200 magnification. Determination of hepatic cholesterol (b) and triglyceride (c) was performed. Data are expressed as mean ± standard deviation (SD). ^##^ *P* < 0.01 compared with naive group; ^*∗*^ *P* < 0.05, ^*∗∗*^ *P* < 0.01 compared with control group.

The high-fat diet induced lipid accumulation in hepatic tissue, as evidenced by multiple and large blanks of lipid droplets in the control group. However, administration of ACE ameliorated these histological alterations (Figure 3(a)). Curcumin exerted an effect similar to ACE.

### 3.3. Hepatic Cholesterol and Triglyceride Content

The high-fat diet considerably elevated hepatic cholesterol and triglyceride serum levels (1.3- and 1.9-fold, resp.). ACE administration significantly lowered hepatic cholesterol (*P* < 0.05 for 200 mg/kg, Figure 3(b)) and triglyceride (*P* < 0.05 and *P* < 0.01 for 100 and 200 mg/kg, resp., Figure 3(c)) levels compared with the control group. The effects of curcumin were similar to ACE.

### 3.4. Serum Lipid Profiles and Glucose Levels

The high-fat diet significantly increased serum TC (1.6-fold), LDL-C (2.1-fold), HDL-C (1.4-fold), triglyceride (1.6-fold), and glucose (1.4-fold) levels. ACE treatment significantly ameliorated these alterations including TC, LDL-C, triglyceride, and glucose levels (*P* < 0.05 or *P* < 0.01 for 100 and 200 mg/kg). Serum HDL-C levels were not altered significantly in ACE-treated groups compared with the control group (Table 2). Curcumin exerted an effect similar to ACE on the profiles of all measured lipids and glucose levels.

### 3.5. Serum and Hepatic Levels of Oxidative Stress Biomarkers

The high-fat diet drastically increased the serum ROS (1.8-fold) and hepatic ROS levels (1.4-fold), malondialdehyde (MDA; 14.5-fold), and protein carbonyl (1.7-fold) compared with the naive group. However, ACE treatment significantly ameliorated the increase of serum ROS (*P* < 0.05 for both) and hepatic ROS levels (*P* < 0.01 for 200 mg/kg), MDA (*P* < 0.001 for both), and protein carbonyl (*P* < 0.05 for 100 mg/kg, Table 3).

The high-fat diet drastically altered the hepatic antioxidant biomarkers such as TAC, total GSH, GSH-Px, GSH-Rd, SOD, and catalase activity in the control group. In particular, total GSH, GSH-rd, and SOD activity were significantly depleted in the control group compared with the naive group, while ACE administration significantly ameliorated these depletions compared with the control group (*P* < 0.05 or *P* < 0.01). The hepatic TAC was not affected significantly by ACE treatment. Interestingly, the high-fat diet induced the GSH-Px increase and catalase activity in the control group, whereas ACE administration augmented their activity without statistical significance (Table 3). Curcumin exerted effects similar to ACE on oxidative stress biomarkers.

### 3.6. Gene Expression Analysis

**Figure 4:**
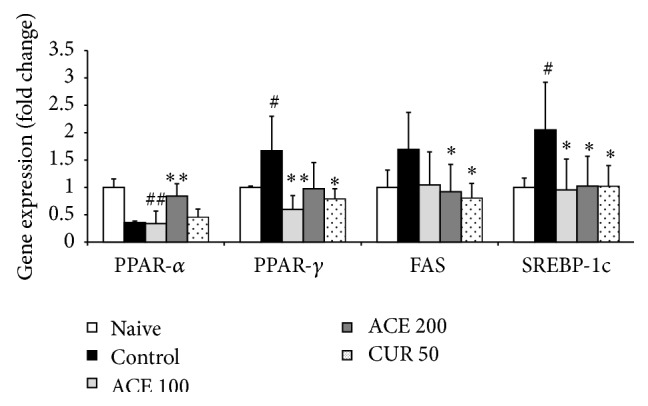
Gene expression levels in liver. Hepatic mRNA expression levels of FAS, PPAR-*γ*, SREBP-1c, and PPAR-*α* were determined using real-time polymerase chain reaction (qPCR). Data are expressed as average ± standard deviation (SD; fold change relative to naive group). ^##^ *P* < 0.01 compared with naive group; ^*∗*^ *P* < 0.05, ^*∗∗*^ *P* < 0.01 compared with control group.

The high-fat diet markedly upregulated the hepatic gene expressions of PPAR-*γ*, FAS, and SREBP-1c, while PPAR-*α* was downregulated. Administration of ACE significantly normalized the gene expression changes compared with the control group (*P* < 0.05 or *P* < 0.01, Figure 4). Curcumin exerted similar effects to ACE.

## 4. Discussion

Metabolic syndrome is the clustering of metabolic abnormalities, such as hyperlipidemia, glycemia, and hypertension in an individual. Diet-induced obesity is a critical etiological factor of theses metabolic abnormalities [21]. Abdominal obesity is a critical factor in the development or precession of various present-day disorders [22]. As expected, a 10-week high-fat diet led to obesity indicated by increased body and peritoneal adipose tissue weights, and ACE treatment significantly inhibited these pathological alterations. Furthermore, the high-fat diet significantly increased serum TC, LDL-C, and triglyceride levels as well as glucose levels, but ACE significantly attenuated these abnormalities in serum lipid profiles and glucose levels.

Hepatic steatosis is considered an important consequence of metabolic syndrome, leading to the subsequent development of necrosis, inflammation, cirrhosis, and hepatocellular carcinoma [23]. In the present study, histological findings and hepatic lipid profiles revealed macrovesicular steatosis in liver tissues, which were reduced significantly with ACE treatment. Furthermore, PPAR*γ*, FAS, and SREBP-1c are proteins involved in the production of lipids in hepatic tissues [24, 25] and PPAR*α* is a key modulator of lipid lysis in the liver [26]. In the present study, the high-fat diet significantly induced upregulation of lipogenic genes (PPAR*γ*, FAS, and SREBP-1c) and suppression of a lipolysis gene (PPAR*α*). ACE ameliorated the altered gene expressions, which supports the antimetabolic abnormality effects of ACE.

Numerous experimental and clinical observations have suggested that oxidative stress is an essential pathogenic component of metabolic syndrome [27, 28]. Overconsumption of fat leads to excessive ROS production, which causes pathological changes in blood vessels, signaling pathways, and inflammation [27]. In the present study, a high-fat diet produced intense oxidative stress in the serum and liver, as evidenced by high levels of oxidants (ROS, MDA, and protein carbonyl) and low levels of antioxidants (GSH-system and SOD). ACE treatments significantly attenuated the altered oxidative stress markers.

The selection of ACE was based on the traditional oriental pharmacological theory of removing “*dampness/phlegm*” (*濕痰*) and “*blood stasis*” (*瘀血*) to treat metabolic syndrome. Many experimental studies have demonstrated antiobesity or antihyperlipidemic effects using the individual herbs* A. iwayomogi *or* C. longa* [8, 10]. One major component of* C. longa, *curcumin, a reference compound, was reported to have anti-insulin resistance and hyperlipidemia effects [9, 29] and exerted similar beneficial effects in the current study. We reported previously the antiatherosclerotic activity of ACE using an apoE knockout mouse model [11], but the present study is the first to demonstrate a pharmaceutical effect of ACE against metabolic syndrome. No adverse event was observed in any experimental groups of ACE and curcumin, respectively.

Together, our results conclusively show the pharmaceutical potential of ACE as an herb-derived remedy improving the metabolic abnormalities. Further studies are necessary to explore the underlying mechanisms of the activities and synergistic effects of ACE by comparing the two compositional herbs.

## Figures and Tables

**Table 1:** Food intake, body, liver, and adipose tissue weights.

Groups	Naive	Control	ACE 100	ACE 200	Curcumin 50
Food intake (g/day/mouse)	2.55 ± 0.54	2.61 ± 0.49	2.94 ± 0.58^*∗∗*^	2.6 ± 0.6	2.65 ± 0.58

Body weight					
Initial day (g)	20.6 ± 0.6	21.1 ± 0.8	20.8 ± 0.97	21.0 ± 0.6	21.23 ± 0.68
Final day (g)	27.1 ± 1.3	40.7 ± 1.4^###^	36.7 ± 1.6^*∗∗∗*^	37.7 ± 1.4^*∗∗∗*^	38.0 ± 1.2^*∗∗∗*^
Liver weight					
Absolute (g)	1.08 ± 0.12	1.68 ± 0.13^###^	1.54 ± 0.12^*∗*^	1.51 ± 0.15^*∗∗*^	1.59 ± 0.07
Relative (%)	3.85 ± 0.49	4.42 ± 0.34^##^	4.11 ± 0.30	3.84 ± 0.42^*∗∗*^	4.01 ± 0.28^*∗*^
Adipose tissue weight					
Epididymal (g)	0.80 ± 0.31	2.83 ± 0.23^###^	2.37 ± 0.49^*∗*^	2.38 ± 0.55^*∗*^	2.42 ± 0.24^*∗*^
Retroperitoneal (g)	0.43 ± 0.23	1.07 ± 0.15^###^	1.03 ± 0.14	0.89 ± 0.18^*∗*^	1.01 ± 0.13
Visceral (g)	0.47 ± 0.18	1.26 ± 0.31^###^	1.17 ± 0.17	1.18 ± 0.15	1.09 ± 0.18
Total (g)	1.80 ± 0.52	5.17 ± 0.41^###^	4.58 ± 0.56^*∗*^	4.46 ± 0.62^*∗∗*^	4.53 ± 0.34^*∗∗*^

Data are expressed as mean ± standard deviation (SD). ^##^ *P* < 0.01 and ^###^ *P* < 0.001 compared with naive group. ^*∗*^ *P* < 0.05, ^*∗∗*^ *P* < 0.01, and ^*∗∗∗*^ *P* < 0.001 compared with control group. ACE (water extract of *Artemisia iwayomogi* and *Curcuma longa*).

**Table 2:** Serum biochemistry parameters.

Groups	Naive	Control	ACE 100	ACE 200	Curcumin 50
TC (mg/dL)	116.9 ± 15.0	182.05 ± 33.86^###^	141.54 ± 6.47^*∗∗∗*^	137.16 ± 17.03^*∗∗∗*^	145.77 ± 14.24^*∗∗*^
LDL-C (mg/dL)	37.3 ± 10.5	79.2 ± 38.6^###^	38.7 ± 10.1^*∗∗∗*^	37.8 ± 10.7^*∗∗∗*^	46.1 ± 29.8^*∗∗*^
HDL-C (mg/dL)	53.5 ± 5.8	74.3 ± 8.8^###^	77.7 ± 8.6	80.00 ± 9.0	85.81 ± 6.7^*∗∗*^
Triglyceride (mg/dL)	102.4 ± 14.1	159.12 ± 15.1^###^	125.56 ± 14.4^*∗∗∗*^	115.5 ± 12.3^*∗∗∗*^	112.9 ± 19.2^*∗∗∗*^
Glucose (mg/dL)	198.3 ± 51.8	281.3 ± 24.9^##^	267.0 ± 39.4	233.3 ± 31.2^*∗*^	225.6 ± 51.4^*∗∗*^

Data are expressed as mean ± standard deviation (SD). ^##^ *P* < 0.01 and ^###^ *P* < 0.001 compared with naive group. ^*∗*^ *P* < 0.05, ^*∗∗*^ *P* < 0.01, and ^*∗∗∗*^ *P* < 0.001 compared with control group. TC, total cholesterol; LDL-C, low-density lipoprotein cholesterol; HDL-C, high-density lipoprotein cholesterol; ACE (water extract of *Artemisia iwayomogi* and *Curcuma longa*).

**Table 3:** Oxidative stress and antioxidant parameters.

Groups	Naive	Control	ACE 100	ACE 200	Curcumin 50
Serum					
ROS (U/mL)	772 ± 88	1,428 ± 402^##^	1,091 ± 211^*∗*^	1,041 ± 368^*∗*^	976 ± 358^*∗*^

Liver					
ROS (U/mg protein)	127.9 ± 26.9	180.5 ± 57.8^##^	166.4 ± 54.1	96.6 ± 20.3^*∗∗*^	109.3 ± 17.8^*∗∗*^
MDA (*μ*M/mg protein)	2.9 ± 1.7	42.1 ± 20.0^###^	10.0 ± 18.2^*∗∗∗*^	5.13 ± 4.5^*∗∗∗*^	2.8 ± 1.2^*∗∗∗*^
Protein carbonyl (*μ*M/mg protein)	50.5 ± 19.3	85.9 ± 21.5^##^	67.3 ± 8.9^*∗*^	74.5 ± 10.8	64.4 ± 10.0^*∗*^
TAC (*μ*M/mg protein)	901 ± 138	648 ± 111^##^	521 ± 128	714 ± 263	550 ± 119
Total GSH (mM/mg protein)	2.31 ± 0.18	1.88 ± 0.17^###^	2.25 ± 0.33^*∗∗*^	1.54 ± 0.44^*∗*^	1.57 ± 0.29^*∗*^
GSH-px (U/mg protein)	8.78 ± 2.71	10.88 ± 3.70	10.62 ± 3.21	14.75 ± 8.11	25.04 ± 7.80^*∗∗∗*^
GSH-rd (U/mg protein)	7.7 ± 1.62	5.82 ± 0.43^##^	6.8 ± 1.20^*∗∗*^	7.23 ± 1.93^*∗*^	5.37 ± 0.87
SOD (U/mg protein)	181.1 ± 30.6	117.1 ± 16.4^##^	143.9 ± 24.8^*∗*^	136.7 ± 14.6^*∗*^	124.1 ± 35.5
Catalase (U/mg protein)	923 ± 99	1,168 ± 195^##^	1,421 ± 630	1,388 ± 455	1,060 ± 244

Data were expressed as mean ± standard deviation (SD). ^##^ *P* < 0.01, ^###^ *P* < 0.001 compared with naive group. ^*∗*^ *P* < 0.05, ^*∗∗*^ *P *< 0.01, and ^*∗∗∗*^ *P* < 0.001 compared with control group. ACE (water extract of *Artemisia iwayomogi *and* Curcuma longa*).
